# Hemi-retinal vein occlusion: Characterizing a rare retinal vasculopathy

**DOI:** 10.4103/IJO.IJO_1712_23

**Published:** 2024-01-08

**Authors:** Yogita Kadam, Pratima Thaku, Anthony Vipin Das, Raja Narayanan, Sirisha Senthil, Brijesh Takkar

**Affiliations:** 1 Department of EyeSmart EMR and AEye, Hyderabad, Telangana, India; 2 Indian Health Outcomes, Public Health, and Economics Research Center, Hyderabad, Telangana, India; 3 Anant Bajaj Retina Institute, Hyderabad, Telangana, India; 4 VST Centre for Glaucoma Care, L V Prasad Eye Institute, Hyderabad, Telangana, India

**Keywords:** Big data, hemi-retinal vein occlusion (HRVO), macular edema, neovascular glaucoma

## Abstract

**Purpose:**

To characterize hemi-retinal vein occlusion (HRVO) in patients presenting to a multi-tier ophthalmology hospital network.

**Methods:**

This retrospective, hospital-based study analyzed 2,834,616 new patients between August 2010 and June 2021. Patients with a clinical diagnosis of HRVO in at least one eye were included as cases. Data were collected using an electronic medical record system. Data were compared to the findings noted in branch RVO (BRVO) and central RVO (CRVO) patients.

**Results:**

HRVO constituted 0.9% (*n* = 191) of all the retinal vein occlusions (RVOs), with the mean age being 60.55 ± 10.14 years. Most patients were male (125, 65.45%) with unilateral (92.67%) affliction. Majority presented during the sixth (31.41%) or seventh (32.46%) decade of life. Most patients reported mild (37.07%) or moderate (27.32%) visual impairment, with vision < 20/200 being less common in HRVO (25.8%) and BRVO (17.2%) compared to CRVO (44.1%) (*P* < 0.00001). Glaucoma was diagnosed and treated in 49 (23.90%) eyes, which was much higher than CRVO (11.45%) and BRVO (5.04%) (*P* < 0.001), though neovascular glaucoma was much less than CRVO (2.9% vs. 9.2%) (*P* = 0.0037). On follow-up, HRVO eyes (12.2%) had lesser vision loss compared to CRVO eyes (13.7%) (this difference does not look very significant to me), though BRVO had the least (9.1%) vision loss.

**Conclusion:**

HRVO is a rare RVO, presenting more in males. It causes less-severe visual impairment compared to CRVO. Large majority of patients with HRVO do not have identifiable systemic risk factors other than age. Preexisting glaucoma was more associated with HRVO compared to other RVOs.

Hemi-retinal vein occlusion (HRVO) is a rare retinal vein occlusion (RVO). It occurs when the superior or inferior retinal drainage is affected.^[1,2]^ The site of involvement in the retinal venous system is different from branch RVO (BRVO) or central RVO (CRVO) and since 1989, attempts have been made to characterize HRVO in comparison to other RVOs.^[3–5]^ Some authors have considered it similar to BRVO,^[6–9]^ while others have grouped it with CRVO^[5,10–15]^ and some others have placed it in between BRVO and CRVO in terms of outcomes.^[16]^ Further, one report indicated it to be similar to CRVO at presentation, while the response to treatment was noted similar to BRVO.^[17]^ Hypertension, coronary artery disease, diabetes mellitus (DM), and peripheral vascular diseases are the associated systemic diseases for HRVO like other RVOs.^[11]^ However, there are reports regarding the possible association of specific ocular factors with HRVO compared to other RVOs.^[18]^

Previous studies like the central retinal vein occlusion study (CVOS) and the branch retinal vein occlusion study (BVOS) described the natural course of these diseases, whereas clinical trials have specifically identified treatment for BRVO and CRVO.^[19–22]^ HRVO has been inconsistently categorized as either BRVO or CRVO in these later studies, which complicates interpretation. Moreover, there is limited literature available on the global incidence of HRVO, likely due to its rarity. The objective of this clinical study is to present a larger dataset on the clinical characteristics, risk factors, and outcomes of HRVO and compare them to those of other types of RVOs. We emphasize the importance of recognizing HRVO as a distinct form of RVO, rather than grouping it together with other types.

## Methods

This retrospective, hospital-based study included all new patients presenting between August 2010 and June 2021 to a multi-tier ophthalmology network located in India. Patients gave a standard consent for electronic data privacy at the time of registration. The study adhered to the Declaration of Helsinki and was approved by the Institutional Ethics Committee. Details of data entry into the electronic medical records (EMR) system have been provided elsewhere.^[23]^ A total of 2,834,616 new patients presented to the tertiary and secondary centers of the hospital network during the study period. Records were screened for patients with an ocular diagnosis of HRVO in one or both eyes. A total of 191 patient records (205 eyes) were identified using this search strategy and were labeled as cases. This data was compared to the EMR data of CRVO, BRVO, and RVO overall.

Demographics, clinical presentation, treatment, outcomes, and risk factors were noted for each case and exported to Excel for analysis. Visual acuity (VA) was classified according to the World Health Organization (WHO) guidelines.^[24]^ Stable vision was defined as less than one-line change on the early treatment of diabetic retinopathy study (ETDRS) scale for best corrected distant VA (BCVA) between the first and the last follow-up. Only patients with more than one follow-up (with or without treatment) were included in the stability of vision analysis.

### Statistical analysis

All descriptive analysis was performed using Microsoft Excel 2018, (Microsoft Corporation, Redmond, WA, USA). Chi-square test (StataCorp. 2015, Stata Statistical Software: Release 14; StataCorp LP, College Station, TX, USA) was used for univariate analysis for detecting differences in the distribution of demographic features between patients with HRVO and the overall patient population.

## Results

### Prevalence

Of the 2,834,616 new cases presenting across the eye care network during the study period, 191 were diagnosed with HRVO in at least one eye, translating into a sample prevalence rate of 0.007%, whereas the overall prevalence of RVO was 0.71%. The proportion of HRVO among all RVOs was 0.90%.

### Age

**Figure 1:**
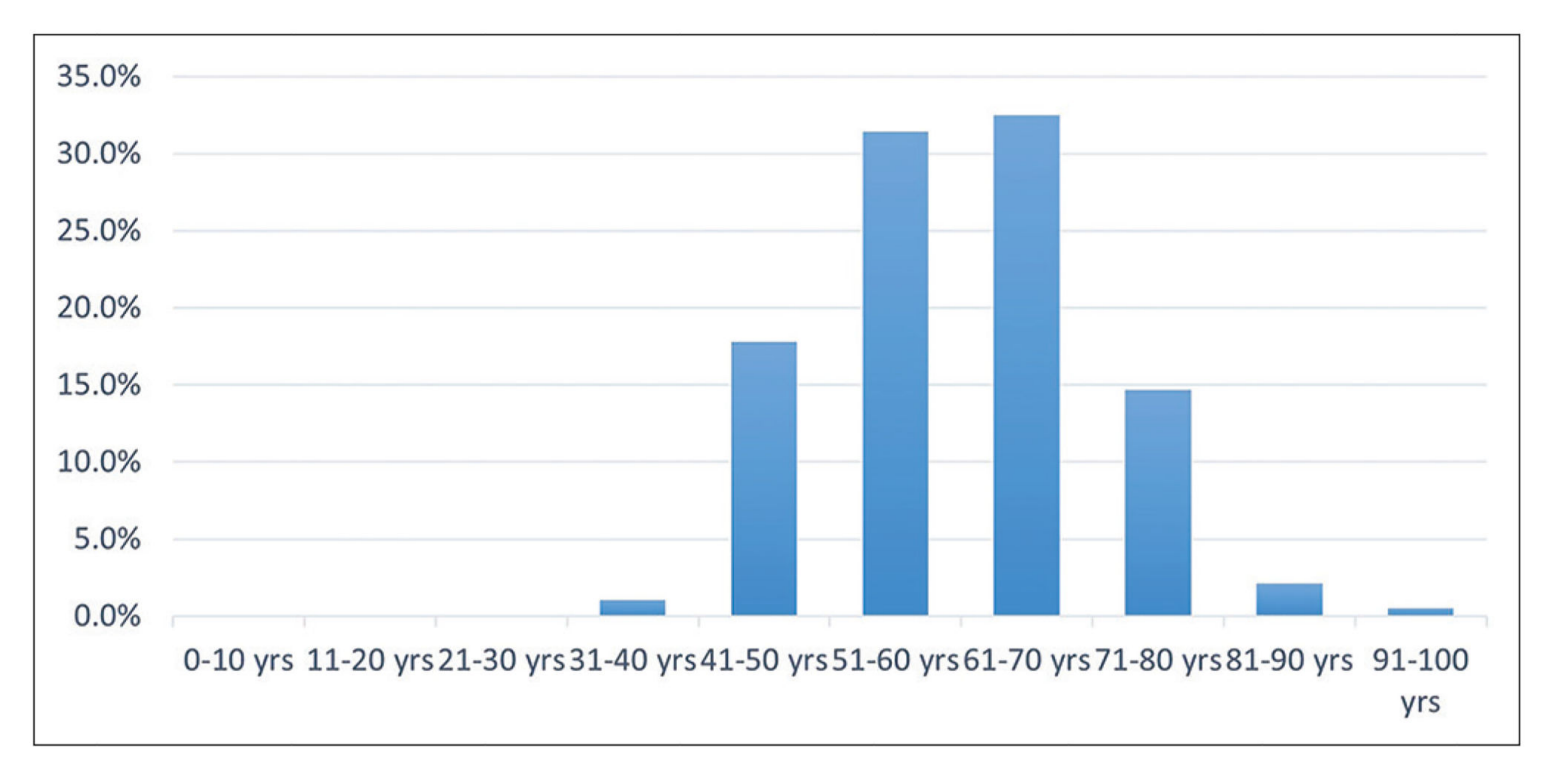
Decade-wise distribution of patients with HRVO. HRVO = hemi-retinal vein occlusion

The mean age of the patients was 60.55 ± 10.14 years, while the median age was 60 years. The mean age of all RVO patients was 58.43 ± 11.90 years. The mean age of patients with BRVO and CRVO was 58.70 ± 11.23 and 56.96 ± 13.40 years, respectively. Most of the patients showed age distribution between 51 and 60 years (*n* = 60, 31.41%) or between 61 and 70 years (*n* = 62, 32.46%) [Fig. 1].

### Gender

There were 125 (65.45%) male and 66 (34.55%) female patients. The overall distribution of HRVO was significantly greater in males (0.008%; 125/1,527,876) compared to females (0.005%; 66/1,306,740) and was statistically significant (*P* < 0.00001). Among the patients diagnosed with HRVO, the mean and median ages were 60 ± 10.01 and 59 (interquartile range [IQR]: 53−67), respectively, in males and 60.98 ± 10.42 and 61 (IQR: 53−68), respectively, in females.

### Laterality

Eighty-seven (45.55%) patients were affected in the right eye and 90 (47.12%) were affected in the left eye. In 14 (7.33%) patients, the affliction was bilateral in nature (any RVO in the fellow eye). Among the 14 patients with RVO in both eyes, two patients had old chronic CRVO in the fellow eye, seven patients had acute CRVO in one eye, and five patients had BRVO in the other eye. Among these, in eight (0.041%) patients, it was a simultaneous presentation (symptoms appeared within 1 month the fellow eye was affected). In these eight patients, one patient had hypertension and DM, two had DM, four patients had only hypertension, and one patient had congenital heart anomaly. The six patients with sequential presentation had no systemic illness.

### Systemic disease

Among 191 patients, hypertension was documented in 38 (19.90%), DM in 32 (16.75%), coronary artery disease in three (1.57%), thyroid disorder in five (2.62%), and cholesterol disorders/dyslipidemia in two (1.05%) patients. Among the patients who visited the hospital between 2020 and 2022, coronavirus disease 2019 (COVID-19) was documented in two (0.000012%) patients.

### Presenting VA

**Figure 2:**
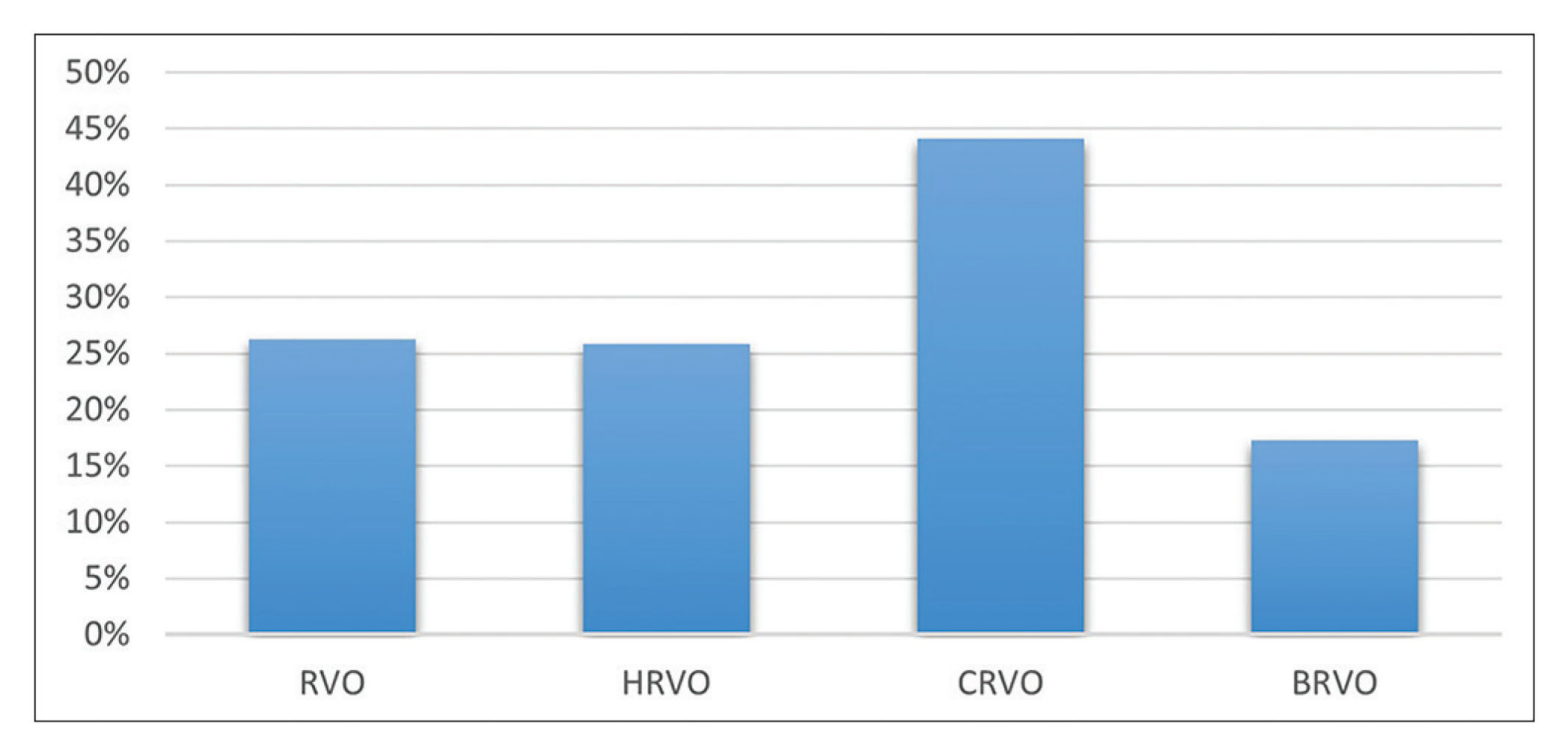
A comparison of VA 20/200 or more in HRVO with CRVO, BRVO, and overall RVO. BRVO = branch retinal vein occlusion, CRVO = central retinal vein occlusion, HRVO = hemi-retinal vein occlusion, RVO = retinal vein occlusion, VA = visual acuity

Of the 205 eyes, 76 (37.07%) eyes had mild or no visual impairment, 56 (27.32%) eyes had moderate visual impairment, and 16 (7.80%) eyes had severe visual impairment. Also, 32 (15.61%) eyes had blindness category 3, three (1.46%) eyes had blindness category 4, and in two (0.98%) eyes, there was no perception of light. In 20 (9.76%) eyes, the best corrected VA was undetermined or unspecified. Moreover, 25.85% of eyes with HRVO had VA 20/200 or worse, while 44.13% of eyes with CRVO, 17.26% of eyes with BRVO, and 26.22% of eyes with RVO overall had VA 20/200 or worse [Fig. 2].

### Last visit VA [Fig. 3]

**Figure 3:**
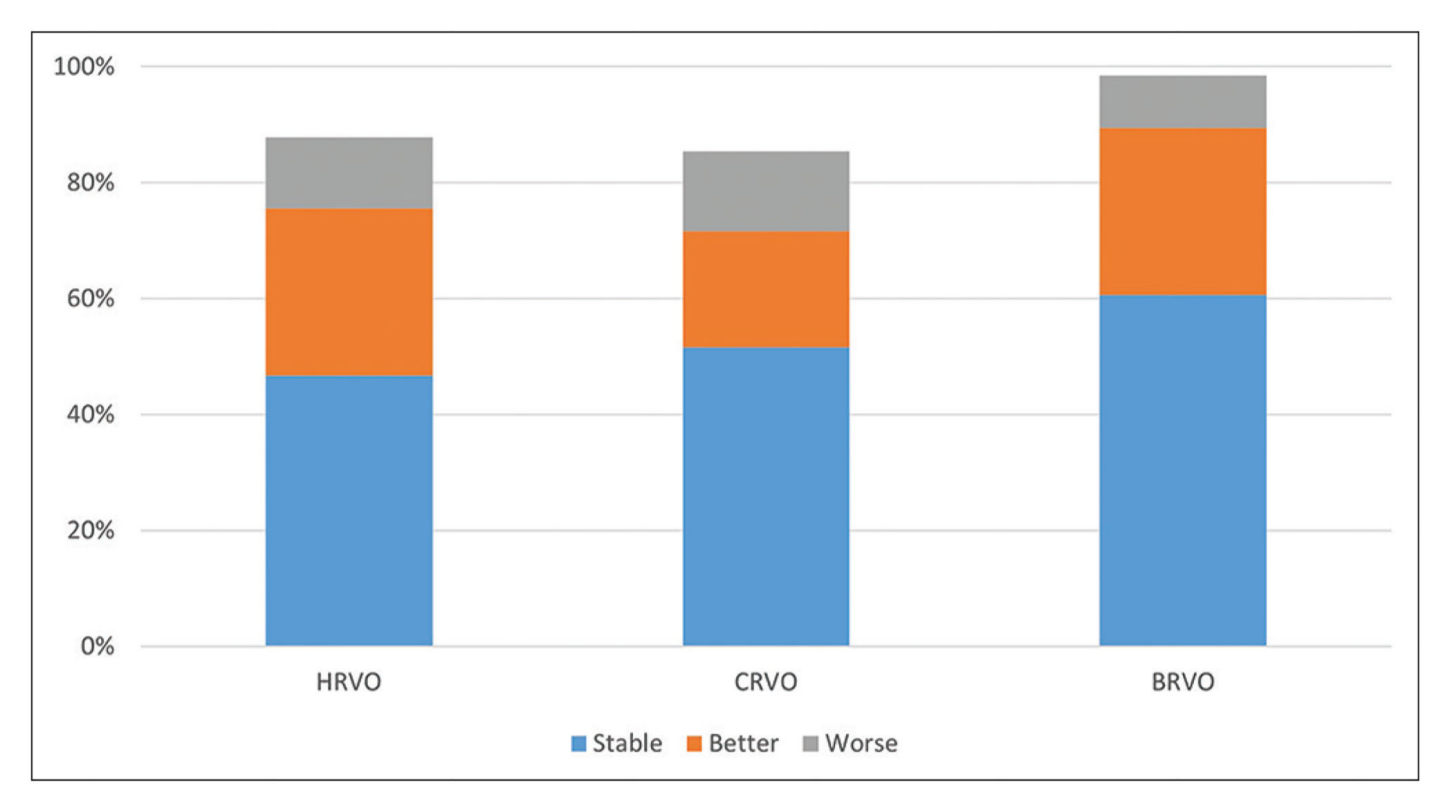
A comparison of improvement in vision in HRVO with CRVO and BRVO. BRVO = branch retinal vein occlusion, CRVO = central retinal vein occlusion, HRVO = hemi-retinal vein occlusion

Of the 205 eyes, 97 (47.32%) had mild or no visual impairment (<20/70), 41 (20.00%) eyes had moderate visual impairment (>20/70−20/200), 22 (10.73%) eyes had severe visual impairment (>20/200−20/400), 24 (11.71%) eyes had blindness category 3, four (1.95%) eyes had blindness category 4 (>20/1200 to PL), four (1.95%) eyes had blindness category 5 (NPL), and in 13 (6.34%) eyes, VA was undetermined or unspecified. More than half the patients had at least two visits (50.78%). The mean number of visits of patients with HRVO was 7.24 ± 7.72 and IQR was 3−9. Of the 205 eyes, 96 (46.83%) eyes had stable vision, 59 (28.78%) eyes had improvement of vision, and 25 (12.20%) eyes had worsened vision from the initial visit [Table 1]. In our CRVO cohort, 51.69% had stable vision, 20.01% had better VA, and 13.69% of eyes had worse VA. In our BRVO cohort, 60.64% eyes had stable vision, 21.37% had better VA, and 9.10% had worse VA (*P* < 0.00001) [Fig. 3]. We also studied the factors associated with change in vision. Glaucoma was seen to be significantly associated with visual loss following HRVO (odds ratio [OR] =2.41, *P* = 0.04), while no other factor showed a statistically significant association [Table 1]. Among 205 eyes, 90 (43.90%) eyes received ocular injections for managing HRVO.

### Ocular comorbidities

#### Glaucoma

**Figure 4:**
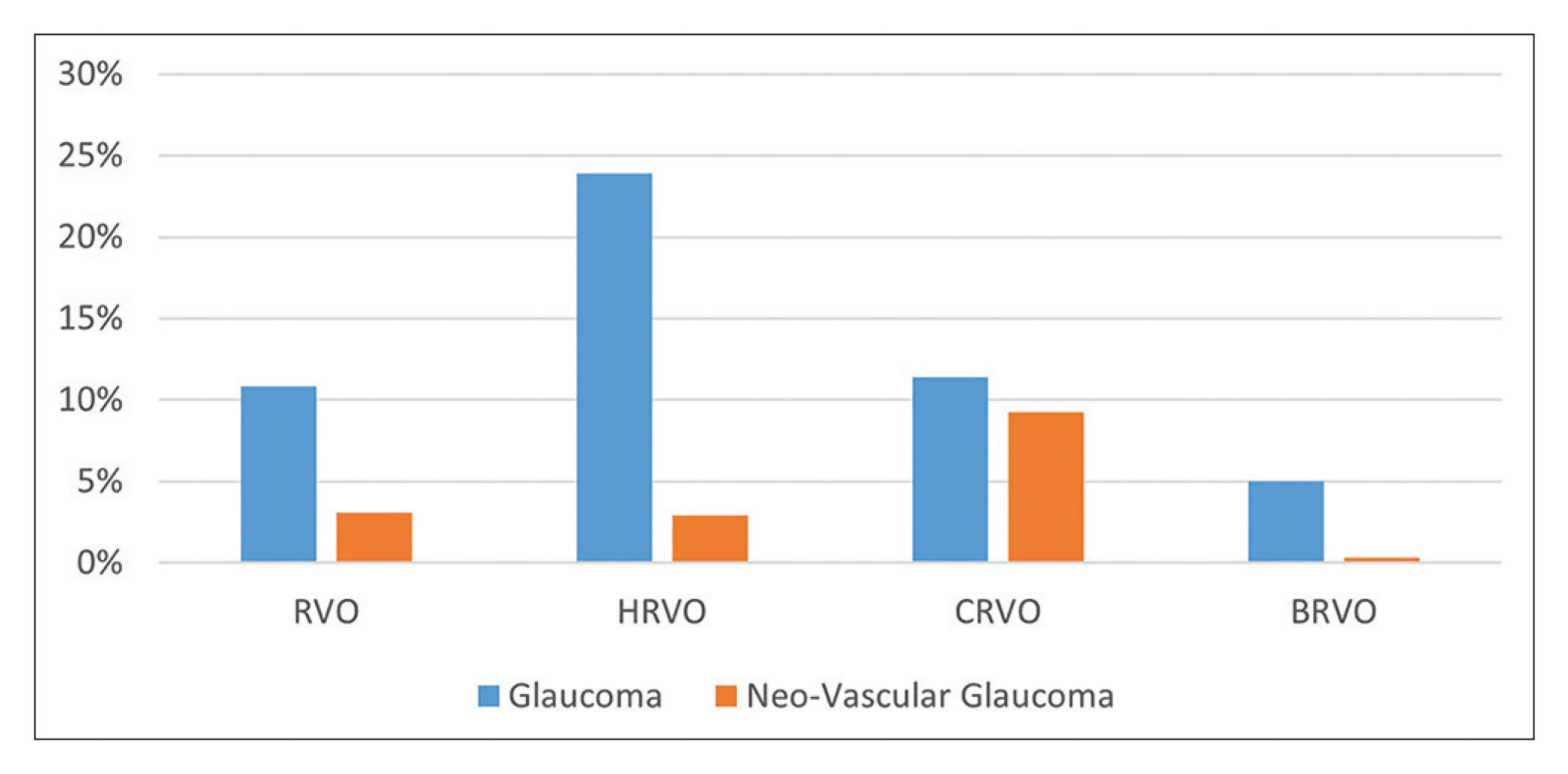
A comparison of glaucoma and neovascular glaucoma in HRVO with CRVO, BRVO, and total RVO. BRVO = branch retinal vein occlusion, CRVO = central retinal vein occlusion, HRVO = hemi-retinal vein occlusion, RVO = retinal vein occlusion

The mean intraocular pressure (IOP; including those on glaucoma medications) in the eyes with HRVO was 15.65 ± 4.96 mmHg (median was 15.56, IQR was 13−17). Glaucoma was diagnosed in 49 (23.90%) eyes, with an OR of 2.0 (95% confidence interval [CI] 0.94−4.21) compared to glaucoma in CRVO. In eyes with CRVO, glaucoma was noted in 11.44% of eyes; in eyes with BRVO, it was seen in 5.04% of eyes; and in overall RVO, it was diagnosed in 10.82% of eyes (*P* < 0.00001) [Fig. 4]. Neovascular glaucoma (NVG) or neovascularization of the iris (NVI) was seen in six (2.93%) eyes in HRVO, 9.27% eyes in CRVO, 0.35% eyes in BRVO, and 3.09% eyes in overall RVO. Out of six HRVO eyes with NVG, four eyes had NVI at presentation and two eyes developed NVI later in follow-up.

The proportion of eyes with HRVO being associated with primary glaucoma was higher compared to other RVOs [Table 2]. More importantly, of the 34 (17.80%) patients with HRVO and glaucoma with completely accessible records, five had DM and/or hypertension (14.7%). Glaucoma involved fellow eyes of 29 of these 34 patients (85.2%). The mean IOP recorded for patients with glaucoma and HRVO (majority on glaucoma medications) was 21.0 ± 7.74 mmHg, the median was 19 mmHg, and IQR was 16−25 mmHg. The highest recorded IOP, whether at our hospital or elsewhere, was <20 mmHg in 17 of these patients (50%), while only three eyes had an IOP >30 mmHg and seven eyes had an IOP >25 mmHg (including the eyes with NVG).

An associated cataract was documented in 52 (25.37%) eyes, diabetic retinopathy in 16 (7.80%) eyes, hypertensive retinopathy in one (0.49%) eye, vitreous hemorrhage in seven (3.41%) eyes, ischemic optic neuropathy in one (0.49%) eye, epiretinal membrane (ERM) in 11 (5.37%) eyes, and optic atrophy in one (0.49%) eye.

## Discussion

HRVO is eight times less prevalent as compared to CRVO and three times less common than BRVO.^[25]^ Our study describes the clinical profile and demographic distribution of HRVO in a large cohort of patients, the largest to date, with an aim to profile the rare disease. The previous largest study to our knowledge documents HRVO in 79 patients.^[26]^ Further, previous evaluations have not presented data on the associated comorbidities and clinical features in detail.^[27]^ Our study shows that glaucoma is an important association of HRVO.

Our results showed that HRVO is seen commonly in the fourth to sixth decade, which is similar to the age of presentation of its counterparts, but with higher male preponderance compared to the other two. All three variants were similar in terms of unilateral presentation being more common than bilateral presentation [Table 3]. It has been recommended that systemic hypertension, DM, and open-angle glaucoma should be part of the evaluation in patients diagnosed with any RVO.^[26,27]^ Similarly, we also found that hypertension (19.90%) and DM (16.75%) were the most commonly documented systemic associations in patients diagnosed with HRVO, while more than half of the subjects did not seem to have any systemic or extra-retinal risk factor for HRVO in our study [Table 2]. Aging-related arterial atherosclerosis and anomalous retinal venous outflow vascular structure may be considered the reasons for such patients.

**Figure 5:**
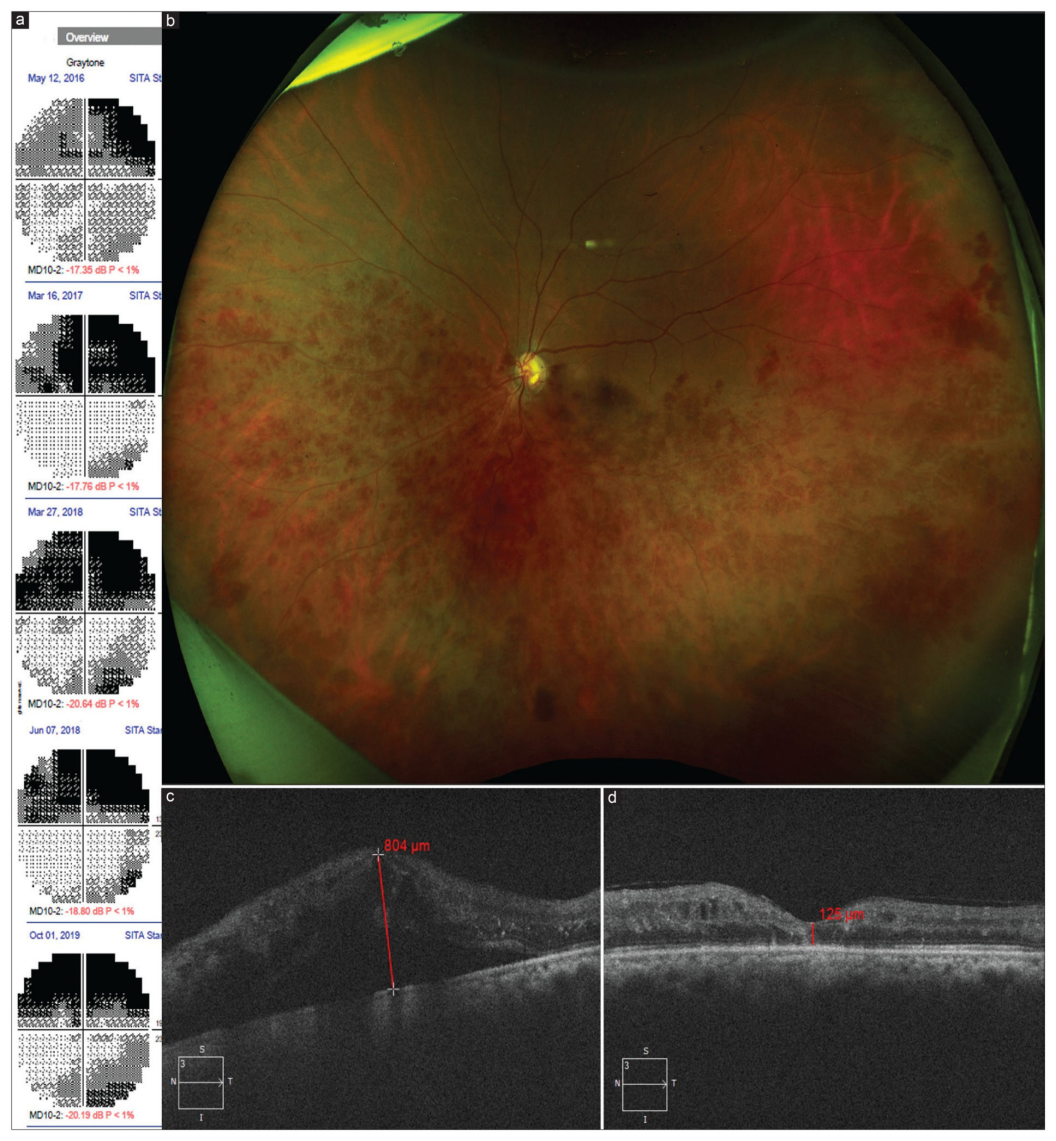
A case representing association of HRVO and glaucoma. A 54-year-old lady with primary angle-closure glaucoma had undergone laser peripheral iridotomy in both eyes and was on topical antiglaucoma medications. LE had presenting visual acuity of 20/25, IOP of 18 mmHg (on medications), and cup-to-disc ratio of 0.8:1, with bipolar notching and biarcuate scotoma in 2016 (a). In the next 3 years, the presenting IOP remained in mid-teens; however, the post-dilated IOP increased to 25−30 mmHg. Repeated 10-2 visual field evaluations of LE showed that the superior scotoma had become denser, while the inferior scotoma developed with time despite stepping up topical medication. In 2019, she presented with drop in visual acuity to 20/160 with IOP in mid-teens and cup-to-disc ratio of 0.9 with a bipolar notch, along with scattered retinal hemorrhages and dilated tortuous retinal vein in the inferior hemifield, prompting a diagnosis of HRVO (b). The vision loss was attributed to macular edema (c), which was treated with intravitreal anti-VEGF agents. Vision recovered to 20/80 and the macular edema subsided (d). HRVO = hemi-retinal vein occlusion, IOP = intraocular pressure, LE = left eye, VEGF = vascular endothelial growth factor

In contrast to these systemic factors, our study found glaucoma as an important ocular association of HRVO. This was twice as common as CRVO and four times more common than BRVO when compared to our own data [Fig. 4]. This trend has also been noted by others [Table 3].^[28–35]^ Hence, glaucoma evaluation should be considered mandatory with repeated surveillance as a part of the clinical assessment of HRVO. We do not have enough data to support the nature of glaucoma, but it is possible that a mechanical change at the optic nerve head predisposes to both HRVO and primary glaucomatous optic neuropathy. In support of this hypothesis, 50% of the glaucomatous eyes with completely accessible records never had an IOP record exceeding 20 mmHg in any hospital and very few (~20%) had IOP recorded in excess of 25 mmHg including NVG eyes [case discussion in Fig. 5].

A causal relationship between COVID-19 and RVO has been a recent question. HRVO diagnosed post-COVID-19 infection in young patients without risk factors has been suggested to be episodic with a favorable prognosis, requiring minimal intervention.^[36]^ It is difficult to answer this question from our data, but the overall prevalence of HRVO was 0.005% in patients who presented between 2010 and 2019 (a 9-year period), while it was 0.011% between 2020 and 2021. This change in pattern of presentation of HRVO can be due to a general change in the pattern of presentation of patients during the pandemic period.

Our study is the largest cohort of patients studied for HRVO to date. The strength of our analysis is the large population size that represents a diverse array of patients. The study does have a few limitations due to its hospital-based method of patient selection, which may have introduced ascertainment bias, but the greatest strength is the complete utilization of the digital data entry in a structured manner by similarly trained ophthalmologists and automated extraction methods for analysis.

In conclusion, our findings show that HRVO is much rarer than other RVOs and affects middle-aged males unilaterally. Not all patients have identifiable risk factors, but simultaneous affection with RVO in both eyes may indicate presence of a systemic risk factor. The association of glaucoma in HRVO may be stronger than in other RVOs. The long-term outcomes of HRVO are worse than BRVO and better than CRVO. HRVO needs to be treated as a separate entity and should not be clubbed with other RVOs during trials.

## Figures and Tables

**Table 1:** Factors associated with visual change in HRVO with unadjusted OR

Factor studied	Better vision	Worse vision
DM	OR=1.16, 95% CI=0.52−2.56, *P*=0.70	OR=1.25, 95% CI=0.43−3.59, *P*=0.67
HTN	OR=0.53, 95% CI=0.23−1.24, *P*=0.14	OR=0.50, 95% CI=0.14−1.79, *P*=0.29
Age>50 years	OR=0.57, 95% CI=0.27−1.19, *P*=0.14	OR=6.42, 95% CI=0.84−49, *P*=0.07
Glaucoma	OR=1.27, 95% CI=0.63−2.54, *P*=0.49	OR=2.41, 95% CI=1.0−5.78, *P*=0.04
Vision at presentation better than or equal to 20/200	OR=1.30, 95% CI=0.69−2.43, *P*=0.40	OR=0.89, 95% CI=0.36−2.22, *P*=0.81
Vision at presentation worse than 20/200	OR=1.02, 95% CI=0.53−1.96, *P*=0.94	OR=1.15, 95% CI=0.45−2.9, *P*=0.75

CI=Confidence interval, DM=Diabetes mellitus, HRVO=Hemi-retinal vein occlusion, HTN=Hypertension, OR=odds ratio

**Table 2:** Comparison of associations of types of retinal vein occlusions

	Hypertenson %	DM %	Age >50 years %	Glaucoma %
BRVO	19.15	16.38	77.07	4.73
HRVO	19.90	16.75	81.15	23.90
CRVO	18.82	25.90	71.32	11.44
RVO	18.33	18.48	76.26	10.82

DM=Diabetes mellitus, BRVO=Branch RVO, CRVO=Central RVO, HRVO=Hemi-RVO, RVO=Retinal vein occlusion

**Table 3:** Comparison of our data with published literature

	HRVO in the current study	CRVO^[6,28−33]^	BRVO^[1,29,31,34,35]^	HRVO in other studies^[17,31]^
Sample prevalence	0.007%	0.1%−0.2%	0.5%−2% (31)	~0.1%
Age (years)	60.55±10.14	61.2±16.7	62±13.1	65−66
Gender	Male > female	Female > male	Female > male	Male > female
Laterality	Unilateral > bilateral	Unilateral > bilateral	Unilateral > bilateral	Unilateral > bilateral
Systemic disease	Diabetes (16.75%) Hypertension (19.90%) Coronary artery disease (1.57%)	Diabetes mellitus 22.9%−36% Hypertension 66%−83.6% Hyperlipidemia 29.8%−35% Metabolic syndrome 37.4% Previous stroke 44% Hypercoagulable state 11.5%−13.6%	Diabetes mellitus 14%−20% Hypertension 65.8%−72.2% Dyslipidemia 17.5% Hyperhomocysteinemia 28%	Diabetes mellitus 11.9% Hypertension 50%−60%
Glaucoma	23.9%	8%−11.4%	5.04%−12.4%	28.6%−40%
Neovascular glaucoma	2.93%	9.27%−33%	0.35%−1.6%	1.6%−1.7%

BRVO=Branch retinal vein occlusion, CRVO=Central retinal vein occlusion, HRVO=Hemi-retinal vein occlusion
